# Supplementary material for: High-resolution genome topology of human retina uncovers super enhancer-promoter interactions at tissue-specific and multifactorial disease loci
Source: Nat Commun. 2022 Oct 7;13:5827. doi: 10.1038/s41467-022-33427-1 (PMC9547065; doi:10.1038/s41467-022-33427-1)
Supplement: Supplementary file 1 — Supplementary Information [file 41467_2022_33427_MOESM1_ESM.pdf]

Supplementary Information

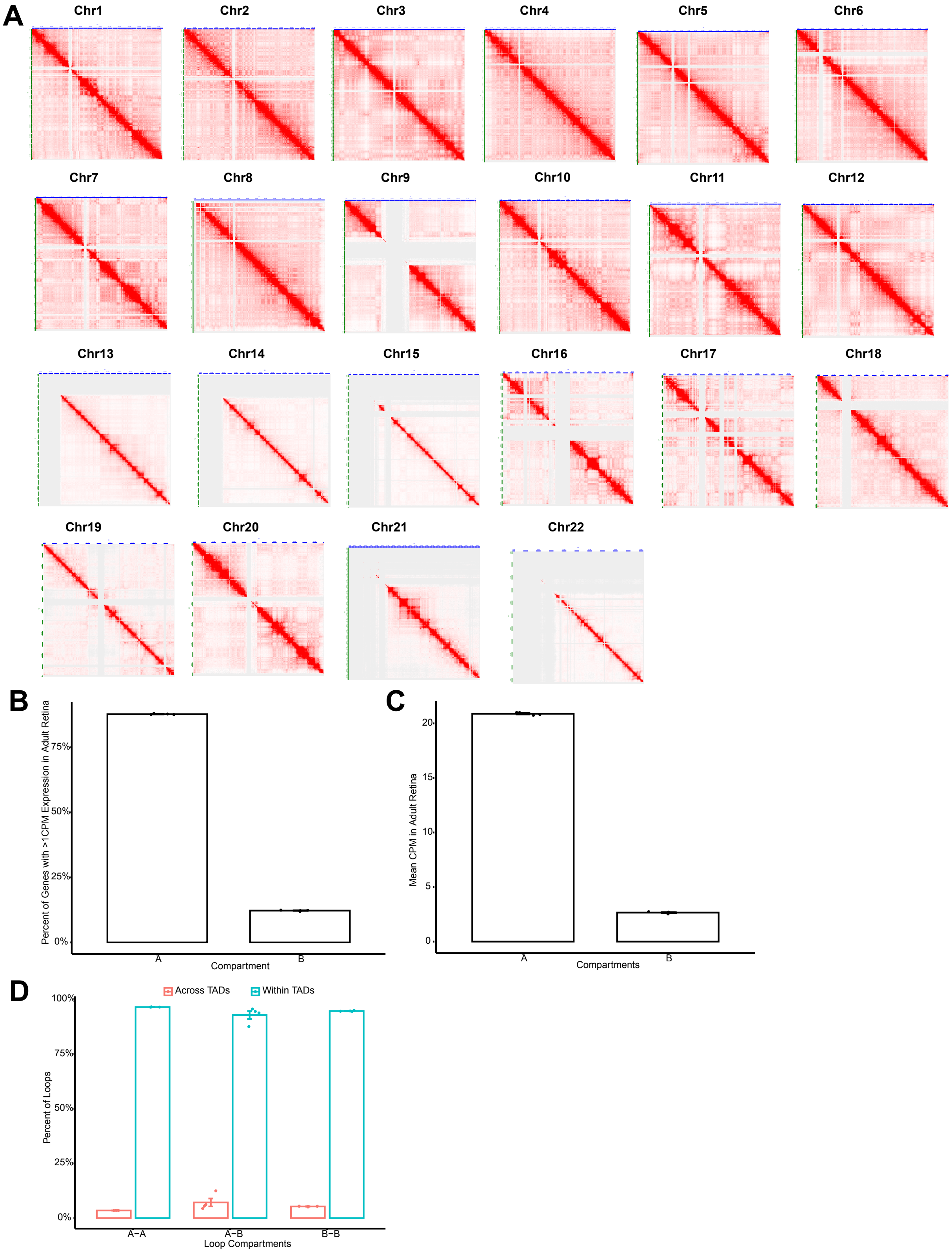

Supplementary Figure 1. Related to Figure 1.

[A] Hi-C contact maps of observed contacts across each autosome. [B] Proportion of expressed gene in A *versus* B retinal compartments. [C] Mean expression of genes in A *versus* B retinal compartments [D] Proportion of intra-TAD versus inter-TAD loops within compartment A (A-A), between compartments A and B (A-B) and within compartment B (B-B). For panels [B-D] n=4 biological replicates, prior to merging datasets. Error bars represent standard error of the mean.

A

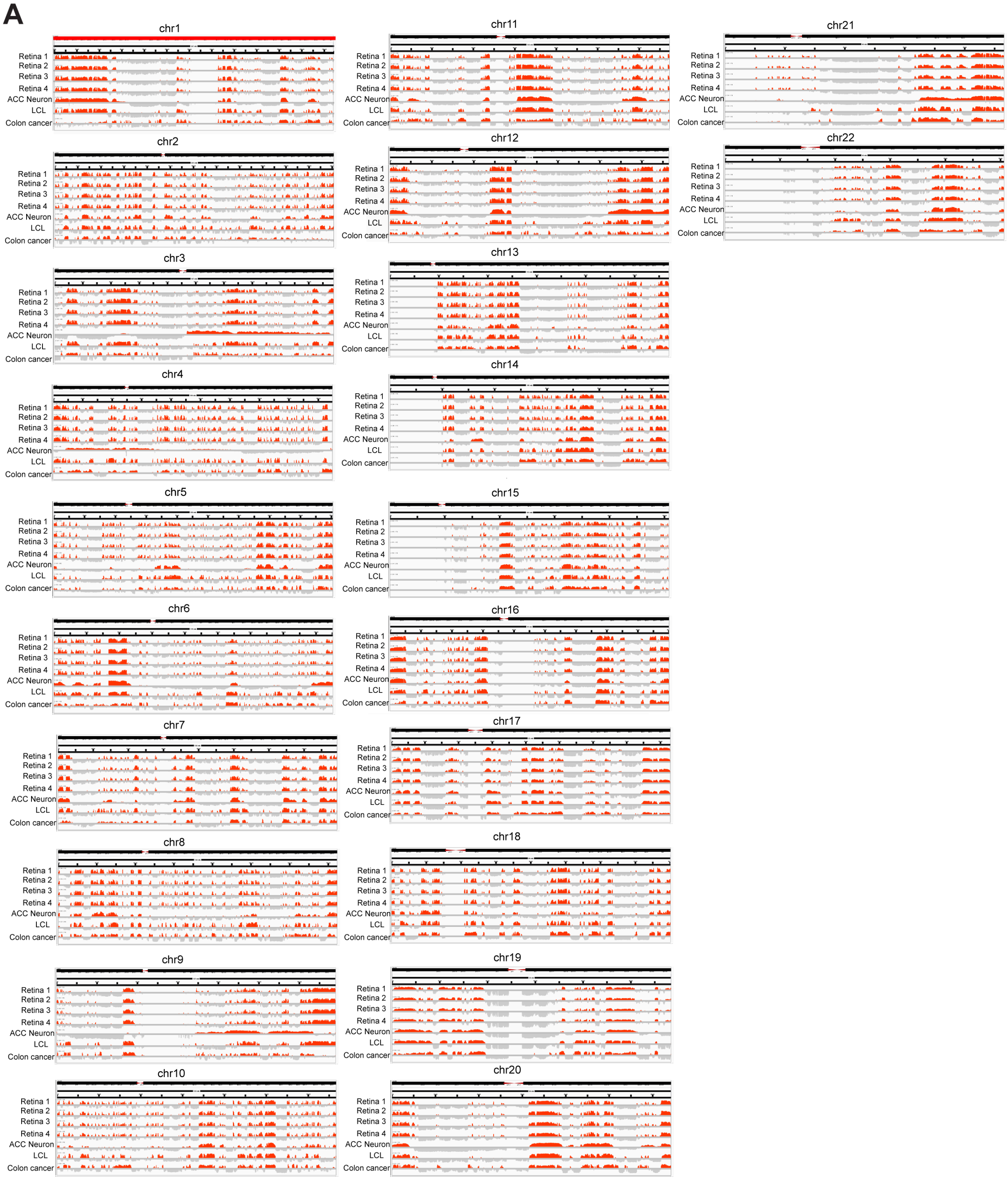

Supplementary Figure 2. Related to Figure 2.

[A] A (red) and B (grey) chromatin compartment represented by the PC1 value on all autosomes for the 4 human retina, the public ACC neurons, the public LCL and the colon cancer Hi-C.

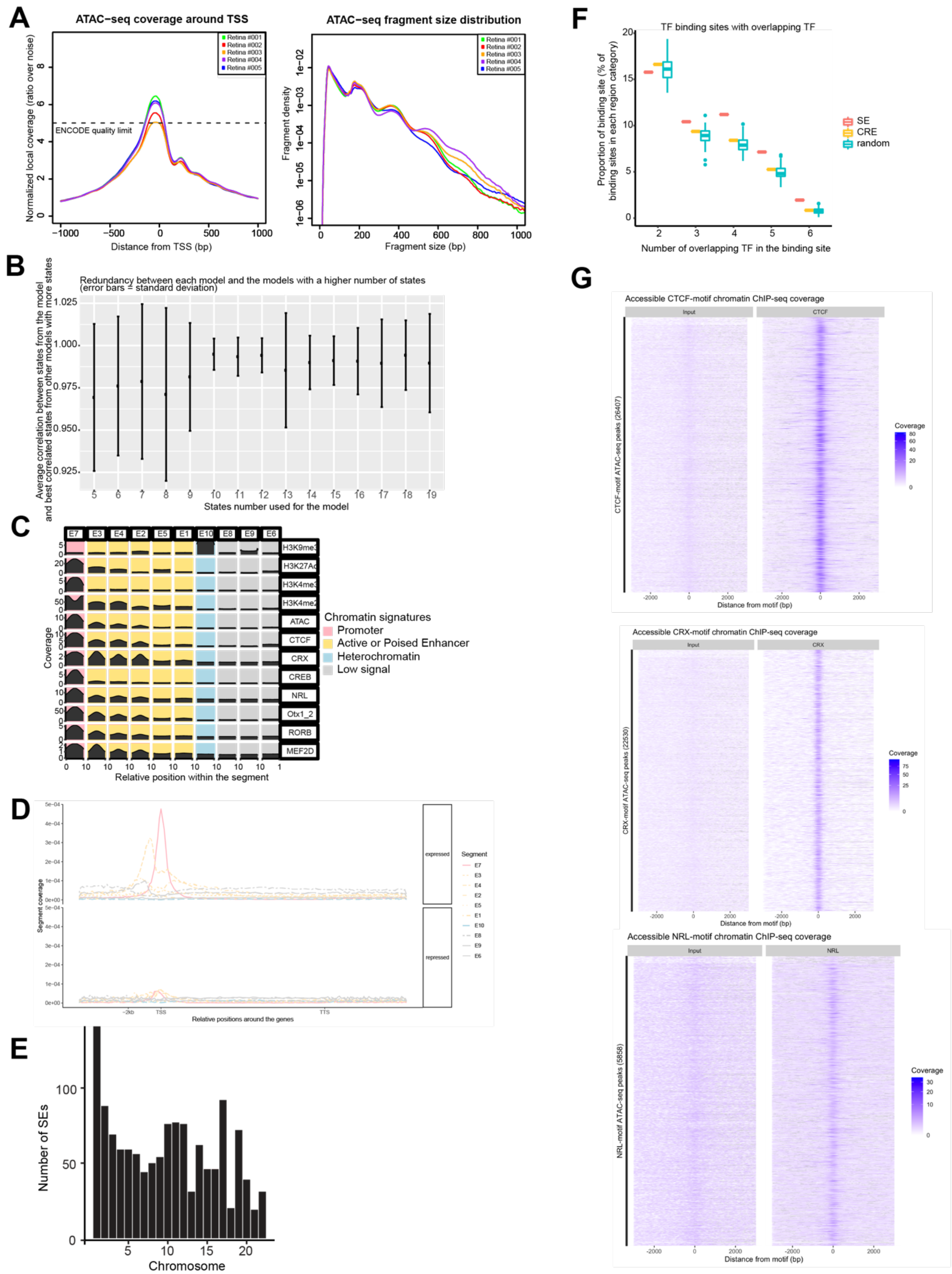

Supplementary Figure 3. Related to Figure 3.

[A] Quality control of the ATAC-seq experiments. Left panel: Normalized coverage around TSS (ratio over the coverage at + / – 800 to 1000 bp from the TSS), grey dotted line represents ENCODE quality threshold. Right panel: ATAC-seq fragments size distribution. [B] Assessment of the best number of chromatin states for the ChromHMM model. ChromHMM has been run using 5 to 20 states generating 16 models. Redundancy between models has been assessed by computing for each model the average correlation between each state from the model and the best correlated state from each model with more states (n=75 correlations for 5 states model, n=84 correlations for 6 states model, n=91 correlations for 7 states model, n=96 correlations for 8 states model, n=99 correlations for 9 states model, n=100 correlations for 10 states model, n=99 correlations for 11 states model, n=96 correlations for 12 states model, n=91 correlations for 13 states mode, n=84 correlations for 14 states model, n=75 correlations for 15 states model, n=64 correlations for 16 states model, n=51 correlations for 17 states model, n=36 correlations for 18 states model, n=19 correlations for 19 states model). Error bars are standard deviation of the mean. Models with > 10 chromatin states show a high redundancy, establishing that 10 states chromatin model is the best to capture different chromatin states from our data. [C] Density plots of histone marks, chromatin accessibility and TF binding for each chromatin state identified by ChromHMM. Each cell indicates the mean coverage for each mark across the segments. Color of each cell represents the manual annotation of the state. [D] Metaprofile showing the density of each chromatin state around expressed and non-expressed genes. Genomic regions of each gene are aligned and resized to overlap the TSS and TTS of the genes. [E] SE distribution across each autosome. [F] Proportion of TF binding sites with 2, 3, 4, 5 or 6 TFs co-bound in SEs, CREs and random regions (n=1 SEs dataset, n=1 CREs dataset, n=100 random regions datasets). Boxplots represent the median and interquartile range (IQR); whiskers mark 1.5x the IQR; data beyond 1.5x the IQR are plotted as individual points. [G] Heatmap of ChIP-seq coverage for Input and CTCF (top), CRX (central) and NRL (bottom).

Each row represents a 5kb genomic region centered on a CTCF (top), CRX (central) or NRL (bottom) accessible motif, ordered by the accessible motif score.

A

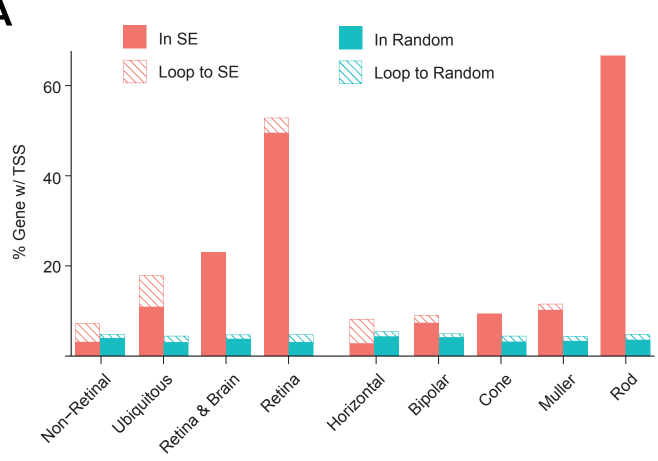

Supplementary Figure 4. Related to Figure 4.

[A] Proportion of genes with the TSS overlapping (plain) or in contact with (hatched) a SE or a random region for different gene categories.

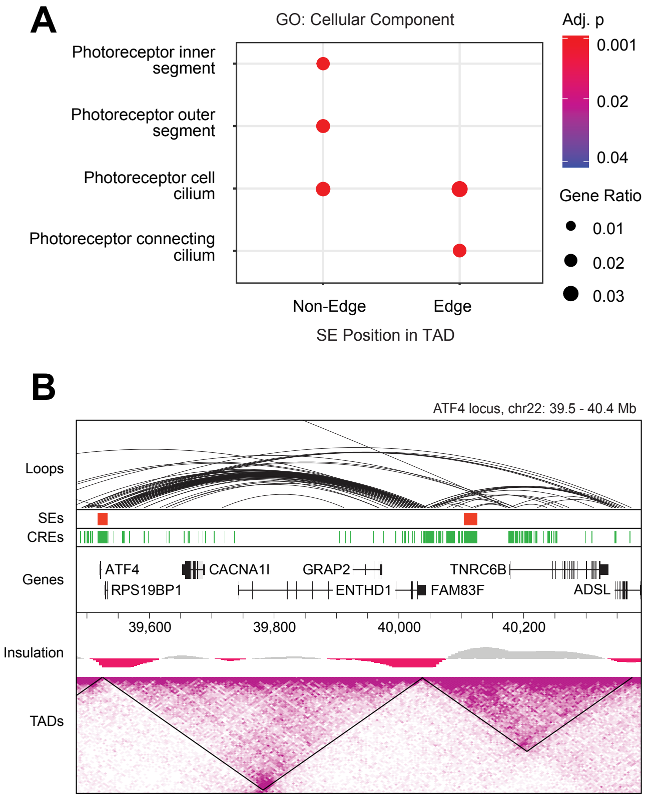

Supplementary Figure 5. Related to Figure 5.

[A] Enriched Cellular Component terms among genes with a TSS residing within or in contact via chromatin looping with a non-edge or edge SE. P values have been FDR corrected. [B] Chromatin loops, SEs, CREs, log_2 insulation score, TADs, and Hi-C contact maps for the *ATF4* locus.

A

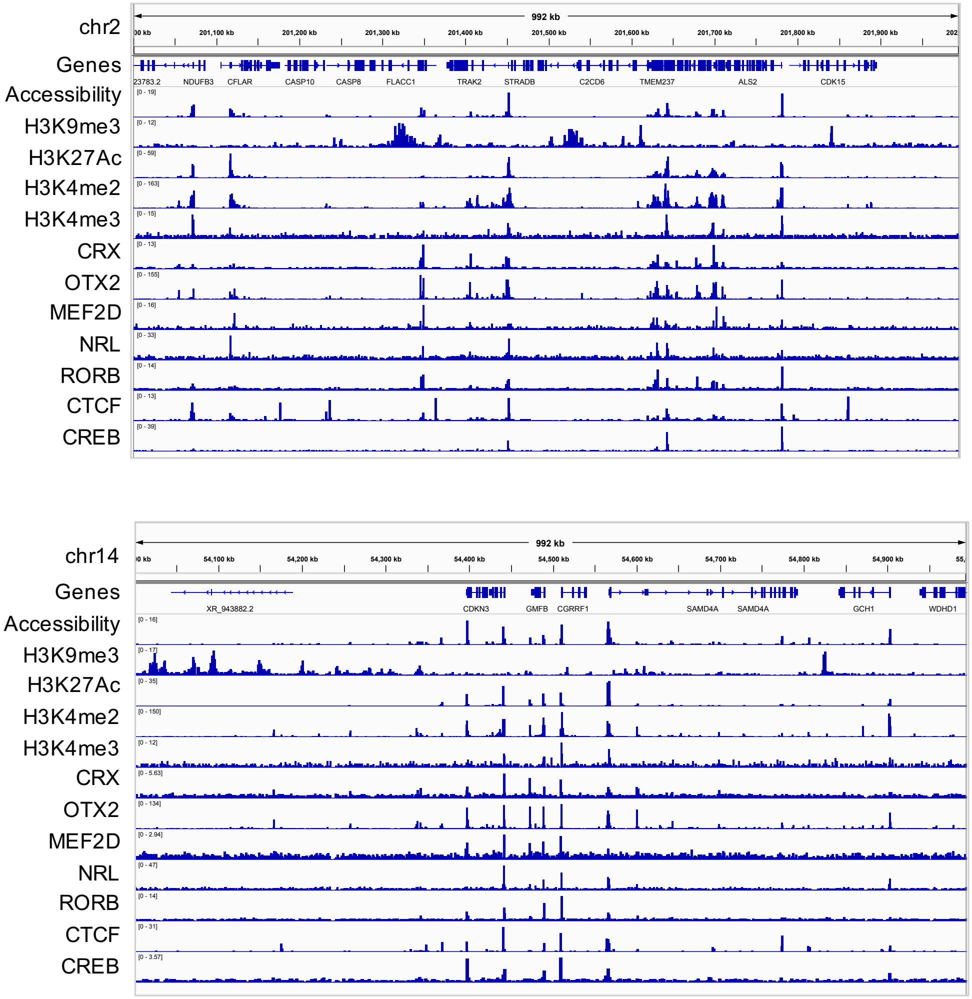

Supplementary Figure 6. Related to Methods.

[A] Genomic coverages for the ATAC-seq, ChIP-seq and Cut&Run data on two representative genomic regions.

Supplementary Table 1

Hi-C statistics summary.

Sample	Read pairs sequenced	Mapped interactions	Valid interactions (% of mapped interactions)	Unique valid interactions	% trans interactions
Retina #1	290,246,313	195,675,050	187,878,052 (96.0%)	182,445,618	23.7
Retina #2	321,806,517	215,009,151	206,389,866 (96.0%)	200,569,059	25.3
Retina #3	283,092,274	190,013,243	180,233,826 (94.9%)	174,190,249	22.3
Retina #4	252,447,626	161,044,850	153,041,279 (95.0%)	147,055,055	23.4
