# Supplementary material for: High-resolution genome topology of human retina uncovers super enhancer-promoter interactions at tissue-specific and multifactorial disease loci
Source: Nat Commun. 2022 Oct 7;13:5827. doi: 10.1038/s41467-022-33427-1 (PMC9547065; doi:10.1038/s41467-022-33427-1)
Supplement: Supplementary file 3 — Description of additional supplementary files [file 41467_2022_33427_MOESM3_ESM.pdf]

Description of Additional Supplementary Files

Supplementary Data 1 : List of genes overlapping a SE, and their expression level in retina.

Supplementary Data 2 : List of TF motifs enriched at the CRX and NRL binding loci that overlap a SE. P value presented have been FDR corrected.

Supplementary Data 3 : List of retinal eQTLs and their associated features (loops, CREs, SEs, nearby genes, genes in contact).

Supplementary Data 4 : List of AMD and glaucoma-associated genomic variants and the linked target genes.
