# Supplementary material for: High-resolution genome topology of human retina uncovers super enhancer-promoter interactions at tissue-specific and multifactorial disease loci
Source: Nat Commun. 2022 Oct 7;13:5827. doi: 10.1038/s41467-022-33427-1 (PMC9547065; doi:10.1038/s41467-022-33427-1)
Supplement: Supplementary file 8 — Reporting Summary [file 41467_2022_33427_MOESM8_ESM.pdf]

nature portfolio

Corresponding author(s): Anand Swaroop

Last updated by author(s): Sep 2, 2022

Reporting Summary

Nature Portfolio wishes to improve the reproducibility of the work that we publish. This form provides structure for consistency and transparency in reporting. For further information on Nature Portfolio policies, see our Editorial Policies and the Editorial Policy Checklist.

Statistics

For all statistical analyses, confirm that the following items are present in the figure legend, table legend, main text, or Methods section.

n/a	Confirmed	
☐	☒	The exact sample size (*n*) for each experimental group/condition, given as a discrete number and unit of measurement
☐	☒	A statement on whether measurements were taken from distinct samples or whether the same sample was measured repeatedly
☐	☒	The statistical test(s) used AND whether they are one- or two-sided<br>*Only common tests should be described solely by name; describe more complex techniques in the Methods section.*
☒	☐	A description of all covariates tested
☒	☐	A description of any assumptions or corrections, such as tests of normality and adjustment for multiple comparisons
☐	☒	A full description of the statistical parameters including central tendency (e.g. means) or other basic estimates (e.g. regression coefficient) AND variation (e.g. standard deviation) or associated estimates of uncertainty (e.g. confidence intervals)
☐	☒	For null hypothesis testing, the test statistic (e.g. *F*, *t*, *r*) with confidence intervals, effect sizes, degrees of freedom and *P* value noted<br>*Give P values as exact values whenever suitable.*
☒	☐	For Bayesian analysis, information on the choice of priors and Markov chain Monte Carlo settings
☒	☐	For hierarchical and complex designs, identification of the appropriate level for tests and full reporting of outcomes
☐	☒	Estimates of effect sizes (e.g. Cohen's *d*, Pearson's *r*), indicating how they were calculated

Our web collection on statistics for biologists contains articles on many of the points above.

Software and code

Policy information about availability of computer code

Data collection	No software was used in the collection of data in this study.
Data analysis	Data analysis in this paper was performed using the following software:<br>bedtools v2.29.2, bowtie2 v2.3.5.1, ChromHMM v1.19, cooltools v0.4.0, cutadapt v3.0, deepTools v3.3.0, domaincaller (no version number provided, downloaded from https://github.com/XiaoTaoWang/domaincaller commit id d410422; August 2020), dplyr v1.0.7, FASTX-Toolkit v0.0.14, FitHiC v2.0.7, GenomicRanges v1.42, ggplot2 v3.3.5, ggpubr v0.4.0, gprofiler2 v0.2.1, HiCRep v1.0.0, HiCUP v0.8.0, HOMER v4.1, IGV v2.11.9, LDlink v5.1, MACS2 v2.2.7.1, Meme Suite v5.4.1 (FIMO and AME algorithms), regioneR v1.26.1, samtools v1.9, R v4.0.3 and v3.6, rgt-HINT v0.13.1, ROSE algorithm (no version number provided, downloaded from https://bitbucket.org/young_computation/rose/src/master/, last commit 27 November 2018) ,ComplexHeatmap v2.7.10.9002

For manuscripts utilizing custom algorithms or software that are central to the research but not yet described in published literature, software must be made available to editors and reviewers. We strongly encourage code deposition in a community repository (e.g. GitHub). See the Nature Portfolio guidelines for submitting code & software for further information.

Data

Policy information about availability of data

All manuscripts must include a data availability statement. This statement should provide the following information, where applicable:

- Accession codes, unique identifiers, or web links for publicly available datasets
- A description of any restrictions on data availability
- For clinical datasets or third party data, please ensure that the statement adheres to our policy

Datasets produced in this study are accessible in GEO under the accession numbers: GSE202471 [https://www.ncbi.nlm.nih.gov/geo/query/acc.cgi?acc=GSE202471]

(Hi-C), GSE202472 [https://www.ncbi.nlm.nih.gov/geo/query/acc.cgi?acc=GSE202472] (ATAC-seq), GSE202473 [https://www.ncbi.nlm.nih.gov/geo/query/acc.cgi?acc=GSE202473] (Cut&Run) and GSE202474 [https://www.ncbi.nlm.nih.gov/geo/query/acc.cgi?acc=GSE202474] (full series). These data can be explored using our user-friendly application on computer, tablet, or smartphone on http://grn.nei.nih.gov. hg38 genome was used for alignment. Gene expression data are from our previous study, under the accession GSE115828 [https://www.ncbi.nlm.nih.gov/geo/query/acc.cgi?acc=GSE115828]. The public ChIP-seq raw data used in this study are accessible under the following SRA numbers: SRR10172858 [https://www.ncbi.nlm.nih.gov/sra/?term=SRR10172858] (H3K27Ac), SRR10172898 [https://www.ncbi.nlm.nih.gov/sra/?term=SRR10172898] (H3K4me2), SRR10172903 [https://www.ncbi.nlm.nih.gov/sra/?term=SRR10172903] (CRX), SRR10172897 [https://www.ncbi.nlm.nih.gov/sra/?term=SRR10172897] (NRL), SRR10172909 [https://www.ncbi.nlm.nih.gov/sra/?term=SRR10172909] (CTCF), SRR10172910 [https://www.ncbi.nlm.nih.gov/sra/?term=SRR10172910] (MEF2D), SRR10172908 [https://www.ncbi.nlm.nih.gov/sra/?term=SRR10172908] (RORB), SRR10172914 [https://www.ncbi.nlm.nih.gov/sra/?term=SRR10172914] (CREB), SRR10172882 [https://www.ncbi.nlm.nih.gov/sra/?term=SRR10172882] (OTX1 / OTX2), SRR10172850 [https://www.ncbi.nlm.nih.gov/sra/?term=SRR10172850] (Input). Public Hi-C data are accessible under the following accession numbers: GSE135465 [https://www.ncbi.nlm.nih.gov/geo/query/acc.cgi?acc=GSE135465] (Mouse retina), Synapse syn12978758 [https://www.synapse.org/#!Synapse:syn12978758] and syn12978762 [https://www.synapse.org/#!Synapse:syn12978762] (Purified neurons) and SRR1658572 [https://www.ncbi.nlm.nih.gov/sra/?term=SRR1658572] (GM12878).

Field-specific reporting

Please select the one below that is the best fit for your research. If you are not sure, read the appropriate sections before making your selection.

☒ Life sciences ☐ Behavioural & social sciences ☐ Ecological, evolutionary & environmental sciences

For a reference copy of the document with all sections, see nature.com/documents/nr-reporting-summary-flat.pdf

Life sciences study design

All studies must disclose on these points even when the disclosure is negative.

Sample size	Five healthy donors (2 females and 3 males) 65-77 years of age at the time of death. The standard for Hi-C studies is 2 to 3 samples per condition. Four postmortem human retina samples were sufficient to obtain at least 5Kb resolution.
Data exclusions	No data were excluded from these analyses.
Replication	Hi-C data was collected from four retina; we confirmed these samples were highly similar as measured by Stratum-adjusted Correlation Coefficient (Figure 2A). We then merged our samples for greater depth in all other analyses. ATAC-seq was performed on five retinal samples, only accessible regions identified in at least three samples were used in our analyses. Cut&Run was performed on only one retina due to limited starting material availability. For Hi-C we have four biological replicates. The success of the technique was determined by performing similar experiment on HCT-116 cell line and flow-sorted mouse rods. All replicates were successful and highly co-related to each other. For ATAC-seq five biological replicates were used. All the replicates were highly correlated. Cut&Run was performed on 3 biological replicates and one passed our stringent quality filter.
Randomization	Our manuscript does not describe an experimental treatment therefore randomization is not a relevant concern. These are post-mortem adult human retina samples that were obtained from an eye bank. The only criteria used for selection was "adult with no eye disease" and how quickly we could get the intact eye after death.
Blinding	Our manuscript does not describe an experimental treatment therefore blinding was not necessary. All the samples included in the study were unidentified postmortem adult retinas with no eye disease.

Reporting for specific materials, systems and methods

We require information from authors about some types of materials, experimental systems and methods used in many studies. Here, indicate whether each material, system or method listed is relevant to your study. If you are not sure if a list item applies to your research, read the appropriate section before selecting a response.

Materials & experimental systems

n/a	Involved in the study	
☐	☒	Antibodies
☐	☒	Eukaryotic cell lines
☒	☐	Palaeontology and archaeology
☒	☐	Animals and other organisms
☐	☒	Human research participants
☒	☐	Clinical data
☒	☐	Dual use research of concern

Methods

n/a	Involved in the study	
☐	☒	ChIP-seq
☒	☐	Flow cytometry
☒	☐	MRI-based neuroimaging

Antibodies

Antibodies used	Antibodies against H3K9me3 (Rabbit, cat.no. ab8898, Abeam, Cambridge, UK), H3K4me3 (Rabbit, cat.no. ab8580, Abeam, Cambridge,

Antibodies used	UK) and control IgG (Rabbit, cat.no. 011-000-002, Jackson ImmunoResearch Laboratories, PA, USA) were used at a concentration of 1:100 in 100ul.
Validation	The antibodies used in this study have the following validation provided via their manufacturers' websites. H3K9me3 (Rabbit, cat.no. ab8898): every new batch of ab8898 is tested via house in Ch IP; suitable for: WB, IHC-P, ICC, ChIP. H3K4me3 (Rabbit, cat.no. ab8580): all batches of ab8580 are tested in Peptide Array against peptides to different Histone H3 modifications; suitable for: PepArr, ChIP, WB, IHC-P, ICC/IF. IgG (Rabbit, cat.no. 011-000-002): Gamma globulins are purified from non-immunized animal serums by salt fractionation, ionexchange chromatography and gel filtration. Gamma globulins are an inexpensive source of IgG with only trace amounts of other immunoglobulins and/or non-immunoglobulin serum proteins.

Eukaryotic cell lines

Policy information about cell lines

Cell line source(s)	HCT-116 (ATCC, Manassas, VA, USA)
Authentication	ATCC authenticated this cell line via Sanger sequencing.
Mycoplasma contamination	Mycoplasma contamination not detected.
Commonly misidentified lines (See ICLAC register)	No commonly misidentified cell lines were used in the study

Human research participants

Policy information about studies involving human research participants

Population characteristics	Five healthy donors (2 females and 3 males) 65-77 years of age at the time of death.
Recruitment	These are post-mortem adult human retina samples that were obtained from an eye bank. As such, these are random samples from unidentified individuals. The only criteria used for selection were "adult" and sample availability.
Ethics oversight	Autopsy materials from unidentified deceased persons is excluded from IRB review and does not require an Office of Human Subjects Research Protections (OHSRP) determination per 45 CFR 46 and NIH policy. OHSRP ID#: 18-NEI-00619

Note that full information on the approval of the study protocol must also be provided in the manuscript.

ChIP-seq

Data deposition

☒ Confirm that both raw and final processed data have been deposited in a public database such as GEO.

☒ Confirm that you have deposited or provided access to graph files (e.g. BED files) for the called peaks.

Data access links *May remain private before publication.*	To review GEO accession GSE202473: May remain private before publication. Go to https://www.ncbi.nlm.nih.gov/geo/query/acc.cgi?acc=GSE202473 Enter token wtcxcmyuldivpyd into the box
Files in database submission	GSM6122982_crG2019-072pf_NDRI-00S-H3K9me3_L7 hg38_main_rmdup_rmblacklist.bw<br>GSM6122983_crG2019-074pf_NDRl-005-H3K4me3_L7 hg38_main_rmdup_rmblacklist.bw<br>GSM6122984_crG2019-075pf_NDRl-005-lgG_L7 hg38_main_rmdup_rmblacklist.bw
Genome browser session (e.g. UCSC)	https://genome.ucsc.edu/cgi-bin/hgTracks?db=hg38&lastVirtModeType=default&lastVirtModeExtraState=&virtModeType=default&virtMode=0&nonVirtPosition=&position=chrX%3A15560138%2D15602945&hgsid= l351337983_uXBUkf7O5CuAnlmWodc9QbeUQ4uc

Methodology

Replicates	1
Sequencing depth	24,998,347 read pairs (H3K9me3), 7,380,768 read pairs (H3K4me3), 2,871,763 read pairs (lgG)
Antibodies	H3K9me3 (Rabbit, cat.no. ab8898, Abeam, Cambridge, UK), H3K4me3 (Rabbit, cat.no. ab8580, Abeam, Cambridge, UK) and control lgG (Rabbit, cat.no. 011-000-002, Jackson lmmunoResearch Laboratories, PA, USA)
Peak calling parameters	No peak calling for the Cut&Run data
Data quality	Sequencing quality has been assessed using fastQC and multiQC. Cut&Run quality has been assessed by HOMER (computing same and different strand enrichments and ratio same / different strand fold enrichment) and by visual inspection using IGV..
Software	Programs used to analyze the Cut&Run data are cutadapt (adapter trimming), bowtie2 (mapping), samtools (mapped reads filtering),

Software	HOMER (coverage computing) and deeptools (bigwig file generation).
